# Facilitating facilitators to facilitate—Some general comments on a strategy for knowledge implementation in health services

**DOI:** 10.3389/frhs.2023.1112936

**Published:** 2023-04-17

**Authors:** Ann Catrine Eldh, Maria Hälleberg-Nyman, Eva Joelsson-Alm, Lars Wallin

**Affiliations:** ^1^ Department of Health, Medicine and Caring Sciences, Linköping University, Linköping, Sweden; ^2^ Department of Public Health and Caring Sciences, Uppsala University, Uppsala, Sweden; ^3^ Faculty of Medicine and Health, School of Health Sciences, Örebro University, Örebro, Sweden; ^4^ Faculty of Medicine and Health, University Health Care Research Centre, Örebro University, Örebro, Sweden; ^5^ Department of Orthopaedics, Faculty of Medicine and Health, Örebro University, Örebro, Sweden; ^6^ Department of Clinical Science and Education, Södersjukhuset, Karolinska Institutet, Stockholm, Sweden; ^7^ Department of Anesthesiology and Intensive Care, Södersjukhuset, Stockholm, Sweden; ^8^ Department of Health and Welfare, Dalarna University, Falun, Sweden

**Keywords:** knowledge implementation, evidence-based practice, guidelines, internal facilitators, leadership, local facilitators

## Abstract

Numerous endeavours to ensure that day-to-day healthcare is both evidence-based and person-centred have generated extensive, although partial, comprehension of what guarantees quality improvement. To address quality issues, researchers and clinicians have developed several strategies as well as implementation theories, models, and frameworks. However, more progress is needed regarding how to facilitate guideline and policy implementation that guarantees effective changes take place in a timely and safe manner. This paper considers experiences of engaging and supporting local facilitators in knowledge implementation. Drawing on several interventions, considering both training and support, this general commentary discusses whom to engage and the length, content, quantity, and type of support along with expected outcomes of facilitators’ activities. In addition, this paper suggests that patient facilitators could help produce evidence-based and person-centred care. We conclude that research about the roles and functions of facilitators needs to include more structured follow-ups and also improvement projects. This can increase the speed of learning with respect to what works, for whom, in what context, why (or why not), and with what outcomes when it comes to facilitator support and tasks.

## Introduction

To sustain safe healthcare with optimal quality, any breach between what procedures should be performed (commended by contemporary evidence) and what procedures are in fact performed needs to be closed (1). Scientists, decision-makers, managers, and clinicians seek support that will enable faster and better uptake of evidence-based healthcare. Consequently, quality improvement and knowledge implementation have progressed over the last decades, efforts that sometimes overlap and other times counteract one another (2, 3). Although resources are now available that aid knowledge implementation, further efforts are needed to understand what really works. Using internal (also known as local) facilitators is encouraged (4), but more knowledge is needed about the engagement, training, and activities of facilitators and the outcomes of such efforts.

A tongue-twister sets the scene: a facilitator facilitates by facilitation. That is, a facilitator is any enabler of knowledge implementation. As such, facilitators come in various forms. For example, a facilitator for the uptake of an innovation could be a contextual element such as staffs’ recognition of the need for and readiness to change routines. The term “facilitator” is also used for interventions dedicated to bridge barriers such as electronic reminders that enable the end-user's uptake and adherence to evidence-based practice guidelines (5, 6). Internal facilitators (IFs), a topic addressed here, are people assigned to facilitate knowledge implementation in their organisations (7–10). That is, IFs help others adopt and sustain the use of evidence-based practices.

Of all implementation theories, models, and frameworks, the integrated Promoting Action on Research Implementation in Health Services framework, i-PARIHS (previously PARIHS) is one of the most prominent when it comes to addressing the IF role and function (11). Yet, a previous analysis found this component infrequently addressed in for example empirical implementation studies (12). As the role of IFs has not received sufficient attention, no single formula exists for appointing, training, and supporting IFs (13). IFs can be and are often selected among staff, serving as singular entities or teams (14). IFs are commended because of their association with their organisations: their experience with and channels to the context where the knowledge implementation shall take place can be beneficial. IFs may be trained and work in different ways and with different approaches (15). Nevertheless, they are charged with promoting change that is supposed to benefit safer and better care. A better understanding of IFs should help implementation science and healthcare efforts. The purpose of this paper is to reflect how engagement and training of IFs, in relation to context elements can affect their activities and outcomes. Further, to present some recommendations for enhancing IFs role and function in knowledge implementation in healthcare.

## Material and methods

In a number of previous and current implementation studies (Table 1), we have promoted IFs as the main vehicle for knowledge implementation. All studies have comprised interviews with the IFs themselves, managers, and fellow staff (with details available in the corresponding papers). Further, all such interviews have been semi-structured, following a similar, semi-structured guide. In this paper, we have applied a hermeneutic approach (24), to shape an overall understanding of the appointment, training, and performance of IFs (including: who were assigned and by whom; how many IFs were allocated; the layout and content of their training and support programs, plus; their activities and experiences).

**Table 1:** Overview of the studies informing this paper.

Research study	IF intervention timepoints	IF training and support	IFs	Type of data collection and analysis to map IFs
Primary leaders implementing stroke guidelines (PLIS) (16, 17)	February–May, 2013	A 4-month program including two workshops (1 day each) and two teleconferences between the workshops (90 min each, after 3 and 6 weeks) delivered to the IF teams.	Rehabilitation first-line managers (physiotherapists, occupational therapists, registered nurses, and physicians).	Individual interviews prior to and after the intervention; analysed with qualitative content analysis (18).
Onset prevention of urinary bladder issues in orthopaedic nursing and rehabilitation (the OPTION pilot (19, 20) and later cluster-randomised trial, addressing urinary incontinence and urinary retention, respectively (21) plus manuscripts in progress)	February–May 2014; May 2021–April 2022	Three full day seminars with all teams and two tele-conferences (separate per site); the ongoing RCT comprises a 12-month support program initiated and concluded in lunch-to-lunch seminars. In addition, monthly conferences via link plus opportunities for exchange via a project-specific website.	Registered nurses, assistant nurses, physiotherapists, and occupational therapists; teams of registered nurses, assistant nurses, occupational- and/or physiotherapists, nursing and rehab first line manager(s).	Individual interviews, prior to and following the intervention; analysed with qualitative content analysis (18).
Managers implementing oral care guidelines for frail older people in nursing homes (MOral) (22)	September–December, 2014	Three half-day seminars and workshops, and two teleconferences.	Registered nurses (first-line nursing managers).	Individual interviews, prior to and following the intervention; analysed with qualitative content analysis (18).
Preference-based patient participation in renal care (dialysis, and predialysis plus dialysis care, respectively) (23) plus manuscripts in progress	October 2019–March 2020	Lunch-to-lunch seminar with all teams; monthly meetings via link: each site team separate or all teams together in accordance with the IF's preferences.	Registered nurses.	Individual interviews, prior to and following the intervention; analysed with qualitative content analysis (18).

## Experiences and discussion

### Internal facilitator recruitment

By default, the appointment of the IFs in our projects was initiated at the point of site inclusion (including the randomisation stage in cases of quasi-experimental or randomised trials). While recruitment of IFs should consider personal characteristics and interpersonal skills and confidence (8), we asked site managers to suggest IFs based on primary commands: assign IFs by means of identifying individuals (a) susceptible to clinical practice change, and (b) with authority among their peers. Consequently, all IFs across the commissioned implementation studies were local staff with an employment within their organisation, recruited by their site manager(s); to the best of our knowledge, none asked their staff to elect an IF. The IFs held typical positions for their professions and were assigned the IF role on a temporary basis for each unique knowledge implementation project. Some had a formal leadership role, and in some cases, both staff and managers were nominated to a local IF team. Many IFs in our cases had some or extensive prior training in and/or experience with quality improvement, but no one had prior knowhow of implementation science.

The IFs in the studies underpinning this paper were all willing to engage in this role. More exactly, most were genuinely keen to engage as IFs and recognised the importance of making changes in their organisation to improve the quality of care. A sense of pride being appointed to facilitate this process has transpired from the very start of each intervention. However, such commitment often diminished over time, regardless of if serving as IFs on a temporary engagement (for the particular implementation project) or having an everyday leadership position in addition to the IF assignment. This is despite the training and support programs offered: all our ventures have addressed the need to plan and perform both in times of tailwind and hardship, including a promotion of strategies to facilitate engagement over time. Rather, we propose such decline of enthusiasm is primarily a matter of focus and local support: it takes time to facilitate knowledge implementation. If—or more often when—other initiatives or requirements for change emerge in the local context, other staffs’ and managers’ attention to and engagement in the project the IF(s) are assigned often diminishes. IFs then seem to find it difficult to persist in what may be a slow pace of adoption and implementation. Particularly, when peers’ interests turn to other novel initiatives, the IFs often waver. Furthermore, ensuring full adoption and addressing resistance to change is complex. This complexity means that the support of management is vital.

We have found that what a manager considers to be a staff's mandate might neither be what fellow staff nor the IFs themselves consider to be their authority. Rather, who has actual authority among peers, to what extent, and in what issues is delicate to detect. So, it is reasonable to propose that an IF rather needs the capacity to “establish and persist” in a given matter—i.e., whatever innovation (such as an evidence-based guideline or tool) the implementation process addresses. Perseverance is necessary for sustaining the often slow processes of knowledge implementation. Yet, it needs to come with an ability to detect if, when, and how an adaptation is needed—e.g., to adjust an implementation strategy if and when discovering it does not address a barrier, or if detecting additional elements of importance to the implementation process. This adjustment requires attention, confidence, and communicative skills. Such competences can be developed in IFs through implementation coaching, although IFs need a set of basic skills on which to build their further training (25).

### Internal facilitator training and support

In our cases, coaching of IFs has comprised training and support programs. Such interventions for IFs lasted between 3 and 12 months. Training sessions focused on the implementation object, either evidence-based clinical practice guidelines and/or clinical tools. Furthermore, the IFs received material to support their and their peers’ learning about the clinical innovation (i.e., the evidence to be implemented). In addition, the training focused on knowledge implementation. Consequently, both implementation experts and clinical experts served on the programs and helped guide the IFs (13). We name these external facilitators, EFs. All the programs (Table 1) suggested the IFs made plans for promoting the realisation of evidence by mapping local barriers and enablers and fitting implementation strategies to address these features. With the Promoting Action on Research Implementation in Health Services (PARIHS; later the integrated-PARIHS or i-PARIHS) framework as a common backdrop, the local context has been highlighted in seminars where the IFs discussed their plans and procedures along with plans to reinforce knowledge-to-action. This has been embedded with respect to their previous know-how of quality improvement.

All the studies also offered the IFs monthly interactions with the EFs, accommodating the IFs’ own learning, making of plans, performance of tasks and actions, as well as addressing shared challenges and experiences (that is, both practical and intellectual support) (8, 11). These interactions were a mix of face-to-face meetings and telephone and/or teleconference support plus some digital support (via e-mail and/or a project specific website). We did not find much variance with regards to these arrangements, but the regular support was typically appreciated. However, we propose a variance occurs with respect to who is invited to these events. In some projects, the IFs were invited to joint meetings that included all IFs, in others we provided a mix of support for each site's IF(s) and joint meetings. In the former, IFs were occasionally unable to attend, but were offered site-specific encounters. In the latter, we invited the IFs to decide whether to have common or individual meetings. We found that early in IF support programs, local barriers and plans reinforcing site events need to be considered. Consequently, some meetings benefit from a joint setup between the intervention sites so the IFs can learn from each other. This common approach is particularly useful when all IFs have started to facilitate implementation. Yet, although each context is unique and therefore requires a tailored implementation, we found that over time the IFs tended to compare themselves with others in a competitive style. Therefore, we suggest a coordinated number of and structure for joint meetings; these meetings should have an explicit agenda that emphasises learning from each other by sharing “what worked” only if accompanied by “in what context”. That is, mutual meetings need to provide transferable experiences and activities. Unless feasible, individual support—i.e., site support—should be considered.

How much training and support IFs need is a critical issue. In previous implementation research, we experienced that longer training (5 vs. 3 days) and 2 years of monthly support (rather than 1 year) did not increase the impact of the IFs who were assigned to promote better incontinence care of frail older people (26). Rather, in a current RCT with an embedded process evaluation in orthopaedic care (Table 1), the IF teams appreciated the 1-year program although they were prepared for the progress to take longer (personal communication). It may take years for an IF support program to ensure guideline adherence with clinical outcome effects (27), emphasising the need to recognise context and sustained support (28).

Adding training and support to IFs to promote knowledge implementation is also a question of when and how the training and support takes place. In general, IF coaching in our studies was hampered by a lack of literature about clinical implementation, and we appreciate the recent publication of handbooks for scholars and clinicians (29, 30), in addition to training manuals (31). This literature should aid seminars and mutual discussions, enabling flipped classrooms and problem-based learning (32); though increasing the opportunities for better training and support of IFs, we recognise the need to have such guidance in one's native language (8). In all our cases, the IFs also needed (and were provided with) training on the clinical objective such as an evidence-based guideline or tool. We also commissioned material to sustain the IFs and other staffs’ learning trajectories. Such needs have either been common, across all intervention sites, or local. Consequently, we have shared guidelines, sources of evidence such as scientific articles, video presentations with tutorials for the clinical issue. Further, we have provided templates for the IFs to assess and evaluate context barriers and enablers, including mapping the extent of evidence-based practice. Although appreciating having been trained with regards to the evidence, the IFs still hesitated to transmit clinical evidence to their peers. Again, we consider this in relation to their authority, which may be hampered by the fact that the IFs have either been a regular member of staff or a manager. In the first case, despite their training in an IF program, it can be difficult to speak with authority to their peers. In the latter, managers have described being dismissed due to their lack of clinical credibility.

Our studies had a limited number of IFs per site attending the intervention programs, although a broader distribution of teaching and training opportunities is proposed (33). Healthcare is often limited in how many people it can dedicate to facilitating change. Therefore, we propose at least introducing other staff and managers to the basics of knowledge implementation such as the enablers and barriers to knowledge implementation. This may encourage peers to engage in mapping their local conditions for knowledge implementation. Furthermore, a basic understanding of implementation strategies can avert obstacles such as resistance to change and deviation; a joint understanding of which efforts are promoted and why (i.e., to bridge everyday barriers) may promote acceptance among staff. We also propose a common, chief understanding of theories, models, or frameworks that recap and support knowledge implementation, which IFs ought to refer to as a result of their training (34). All IFs have attested that they wish they had known this before: this command would have saved them time and efforts when engaged in and/or promoting quality improvement.

### Barriers and enablers for internal facilitators

In our cases, the IFs were primarily selected among the nursing and/or rehabilitation staff even though all cases required that physicians adopt evidence, such as guidelines and/or tools. In all four cases revisited, first-line managers were involved at some point. Even with some prior formal training in change management (either received as part of a leadership program or quality improvement courses), few first-line managers associated leadership with a facilitator role. Rather, most IFs had only through our programs learned about different leadership styles related to promoting change. Further, the IFs linked their own behaviours to facilitating knowledge implementation only after being guided to such connection (35). None of the IFs recognised any previous support in change-oriented leadership behaviours.

Although all IFs were selected because of their presumed capacity to promote change and hence enable knowledge implementation, they had few opportunities to address beliefs and routines of their peers and/or managers. Rather, when encountering such barriers, the IFs required support from their managers (36). Such support requires that all managers understand the basic principles of implementation processes and can both trust and envision change (37). We have found that IFs without the support of managers and managers without staff IFs on their team can only reach a certain point. Thus, we suggest that IF teams consist of both staff representatives and their first-line managers (including physician representatives when influenced by a change). Teams with representatives from both staff and management can better understand what is facilitating knowledge implementation, and what leadership facilitates change (38). The latter incorporates enabling the IFs to proceed and helps sustain the implementation through strategic performance of task-oriented, relations-oriented, and/or change-oriented behaviours (39).

### Internal facilitators activities and performance

All our cases have promoted an elementary Plan-Do-Study-Act process to guide the IFs while progressing in their knowledge implementation assignments. This is similar to for example the Veteran's Affairs Implementation Facilitation Training (31), likewise stressing the planning phase, and recommending a thorough assessment of the context elements that may or may not pose barriers and enable change, respectively. Drawing on the i-PARIHS framework (11), we have also employed the Ottawa Model of Leadership Implementation, including leadership behaviours promoting knowledge implementation. In addition, we have used the Consolidated Framework for Implementation Research (CFIR) to illustrate the implementation process (40). Moreover, both i-PARIHS and CFIR has served as means for capturing context and the elements and domains that can hinder or enable knowledge implementation. Furthermore, we believe that this should proceed by tailoring strategies to fit local facilitation needs, launched only when an appropriate implementation plan is in place. Nevertheless, we found that many IFs favour known methods of knowledge dissemination such as lectures for all staff (41). Early in an implementation process, these traditional approaches can help address the need for a shared understanding of the objective of the implementation (e.g., a clinical guideline) and the need for change. However, lectures in isolation only marginally facilitate adoption and sustainment of the change needed to embrace new or different procedures. Furthermore, teaching sessions do not facilitate de-implementation of what is no longer a best practice (42). We suspect the promotion of lectures for knowledge implementation is linked to a lack of grit and motivation for change, which is found in many healthcare organisations. A more careful initiation and completion of improvement initiatives is likely beneficial, including an evaluation of what has previously worked (or not), providing for more tailored knowledge implementation.

Mapping the context for barriers and enablers was important for all IFs we have trained and supported. In most cases, mapping has enabled both an understanding of upcoming impediments to change and why prior quality improvement efforts were or were not successful. However, even with barriers identified, we found that some obstacles are not addressed in the further implementation process. Quite often this relates to attitudes among peers and/or management, particularly a resistance to change in general or an unwillingness to adhere to a particular guideline or procedure. IFs propose that they cannot address such beliefs or behaviours regardless of whether they represent a staff or manager IF. Indeed, it is difficult to enable implementation whenever some or even several staff (or managers) resist the promotion of evidence-based practice. So far, we have promoted liaisons with key co-workers, also known as champions (43). We have also encouraged the IFs to adopt task-, relations-, and change-oriented behaviour, a focus that requires IFs to carefully assess their context and employment and evaluate their own actions with respect to source and outcomes (4) (This is certainly the case also for those of us serving as external facilitators.). Nevertheless, people are on their own trajectories when presented with and anticipating change (44), making facilitation a question of employing strategies that comprise a reasonable amount of coercion and enthusiasm. When implementing new guidelines, tools, etc. the IFs also need to prepare staff to address concerns raised by patients (or their next of kin) as a result of new healthcare routines and procedures. This way only, both evidence-based and person-centred healthcare services can transpire.

### Co-design and co-production

Facilitating knowledge implementation should involve all end-users. Although recommended (45), patients were not engaged as IFs or placed on the IF teams in any of our cases (perhaps due to our programs not emphasizing this perspective). This may also be an effect of the often swift events taking place, by a lack of recognition of patients as resources, or not associating patient participation with opportunities for improvements (46). In the projects presented here, the IFs shared some previous experience of collaborating with patients (or their next of kin) in quality improvement initiatives. Since our training and support programs have not addressed potential patient facilitators or their representation on the IF teams, we consider this a topic for future development: any change in clinical practice that encourages the uptake and performance of previously not used but better knowledge and routines will require changes in attitudes and behaviours of individuals, teams, and entire organisations. Consequently, end-users like patients will be affected and therefore should be engaged (47). We suggest adding such elements to implementation and evaluations, investigating if and how co-design and co-production with patients facilitates knowledge implementation.

### Contribution to the field

Knowledge implementation is crucial to evidence-based healthcare, but making changes is complicated when it comes to disseminating and adopting clinical guidelines, tools, and procedures. We suggest that IFs are key to such processes as they have the benefit of knowing their organisations and the people who need to be engaged to ensure knowledge implementation (and de-implementation). Furthermore, IFs have opportunities to adapt to the local context and translate the objectives and activities to others.

This paper adds to the growing understanding of who IFs are, their training and support, and what purpose they serve. Lessons learned include the importance of a careful recruitment process, and sustaining a long-term commitment to knowledge implementation, as outlined in Table 2.

**Table 2:** Overview of the overall understanding of facilitating facilitators to facilitate.

Lesson learned
Recruit internal facilitators (IFs) with caution, recognising their ability to reach out to fellow staff, management, patients, and other stakeholders of the knowledge implementation.
Identify IFs level of perseverance and ensure that it is sustained at times of high resistance to knowledge implementation and change
Certify that IFs map the barriers and enablers for knowledge implementation of the local context, and plan (and reschedule) actions to address these
Execute training and support programs for IFs with booster doses
Engage management to facilitate the facilitators in their facilitation of knowledge implementation

Based on a summary of four studies and additional literature, we suggest that IFs best work in teams. In addition, such teams should engage staff representatives, managers, and presumably end-users such as patient representatives. The activities, performance, training, and support of IFs are complex issues that need further attention in implementation science and practice. Moreover, to the best of our understanding, IFs need more than just training and support. Rather, further attention when it comes to what IFs do and how much time they spend on knowledge implementation is needed.

Based on our experience, a more careful engagement of professionals is needed to scaffold a solid and sober plan for knowledge implementation that sustains short- and long-term change while accommodating new needs as they arise. Although IFs with optimal features, prospects, and training are likely to facilitate implementation of evidence-based practice, further investigations into their collaboration with patients are needed. For example, future studies should investigate how to co-design knowledge implementation with patient representatives, focusing on if and how patients can facilitate more evidence-based and person-centred health services.

## Data Availability

The data analyzed in this study is subject to the following licenses/restrictions: Data identifying individuals are not available due to research ethics regulations, but details sufficing the Commentary are available upon reasonable request. Requests to access these datasets should be directed to ann.catrine.eldh@liu.se.
